# The ADRENAL score: A comprehensive scoring system for standardized evaluation of adrenal tumor

**DOI:** 10.3389/fendo.2022.1073082

**Published:** 2022-11-24

**Authors:** Xiaochen Zhou, Xuwen Li, Bin Fu, Weipeng Liu, Cheng Zhang, Yu Xia, Honghan Gong, Lingyan Zhu, Enjun Lei, Joshua Kaplan, Yaoliang Deng, Daniel Eun, Gongxian Wang

**Affiliations:** ^1^ Department of Urology, The First Affiliated Hospital of Nanchang University, Nanchang, Jiangxi, China; ^2^ Department of Radiology, The First Affiliated Hospital of Nanchang University, Nanchang, Jiangxi, China; ^3^ Department of Endocrinology, The First Affiliated Hospital of Nanchang University, Nanchang, Jiangxi, China; ^4^ Department of Anesthesiology, The First Affiliated Hospital of Nanchang University, Nanchang, Jiangxi, China; ^5^ Department of Urology, Temple University Hospital, Philadelphia, PA, United States; ^6^ Department of Urology, The First Affiliated Hospital of Guangxi Medical University, Nanning, Guangxi, China

**Keywords:** adrenal tumor, treatment, surgical risks, scoring system, the ADRENAL score

## Abstract

**Objectives:**

To propose an original and standardized scoring system to quantify the functional and anatomical characteristics of adrenal tumor.

**Materials and methods:**

Four groups of consecutive adrenalectomies (n = 458) with heterogeneity in tumor characteristics and surgical approaches, including 212 laparoscopic cases (Group 1) and 105 robotic cases (Group 2) from The First Affiliated Hospital of Nanchang University, 28 robotic cases from Temple University Hospital (Group 3) and 113 laparoscopic cases from The First Affiliated Hospital of Guangxi Medical University (Group 4). All patients were followed up for 4.5 to 5.5 years. Six parameters including functional status or suspicion of malignancy, tumor size, relationship to adjacent organs, intratumoral enhancement on CT, nearness of the tumor to major vessels and body mass index were assessed and scored on a 0, 1 and 2 points scale. Correlation between the sum of the 6 scores and tumor laterality (ADRENAL score) verse operative time (OT), estimated blood loss (EBL), perioperative complications, transfusion, conversion and length of hospital stay was analyzed.

**Results:**

ADRENAL score was a strong predictor of both OT and EBL in all four groups (p < 0.05 for all tests). In Group 2 and 4, higher ADRENAL score seemed to correlate with longer hospital stay. No statistically significant correlation between ADRENAL score and complication, transfusion or conversion was noted yet.

**Conclusions:**

ADRENAL score appears to be a valid predictor of surgical outcomes. It may provide a common reference for adrenal surgery training program, preoperative risk assessment and stratified comparative analysis of adrenal surgeries *via* different techniques and approaches.

## Introduction

The overall frequency of adrenal tumors is as high as 8.7% on autopsy (1) with adrenal incidentaloma being reported in 4% of patients receiving abdominal CT scans (2, 3). While small non-functioning incidentaloma (< 4 cm) with benign appearance can be managed by active surveillance, adrenal lesions that require surgical resection still spans a large spectrum of benign and malignant indications (4). Since the initial report of laparoscopic transperitoneal (5) and retroperitoneal adrenalectomy (6), minimally invasive surgery has been popularized and become the standard of care for benign adrenal lesions (7). With the increase in experience and introduction of da Vinci robotic system (8), even large (up to 8 cm) (9) or organ-confined malignant adrenal tumor can be safely removed *via* minimally invasive technique with comparable outcomes (10).

The functional status and diversity in size and pathology make the adrenal surgery a unique challenge for both urologists and anesthesiologists. The functional status (11), pheochromocytoma (12) and malignancy (13), tumor size (14) and body mass index (BMI) (15) are documented risk factors for adrenal surgery. However, the characterization of these factors is currently descriptive and lacks standardization and integration. Beyond these established risk factors, other anatomical features of the adrenal tumor are routinely considered in surgical planning. In particular, the relationship between the tumor and adjacent organs or major vessels may greatly affect the resectability of the lesion and contribute to surgical risks. A comprehensive scoring system integrating these established risk factors and anatomical features may help in evaluating the difficulty of surgery and assessing intraoperative risks. It may also provide a common reference for training programs and stratified analysis of adrenal surgeries *via* different techniques and approaches.

The objectives of this study were (a) to propose a standardized and original scoring system of adrenal tumors (designated as the ADRENAL Score) for preoperative assessment based on routinely performed endocrinological, oncological and radiological studies; (b) and to evaluate the correlation between the ADRENAL Score verse surgical outcomes and complications.

## Materials and methods

### Patients and tumors

Four groups of patients (n = 458) treated with minimally invasive adrenalectomy were evaluated retrospectively or prospectively. Data of Group 1 consisting of 212 consecutive laparoscopic retroperitoneal adrenalectomy cases performed by Dr.Wang’s team from January 2010 to December 2014 was retrospectively evaluated. Data of group 2 consisting of 105 consecutive robotic retroperitoneal adrenalectomy cases performed by the same surgical team from February 2015 to June 2016 were prospectively collected and evaluated. Group 3 consisting of 28 consecutive cases of robotic transperitoneal adrenalectomy cases performed by Dr.Eun’s team from January 2014 to April 2016 and Group 4 consisting of 113 laparoscopic retroperitoneal adrenalectomy cases performed by Dr.Deng’s team from January 2014 to December 2016 was retrospectively evaluated. All patients were followed up for 4.5 to 5.5 years. Patient demographics in each group were listed in **Table 1**. In summary, 74 (34.91%), 46 (43.81%), 16 (57.14%) and 47 (41.59%) patients were male in Group 1 to 4, respectively. The average age was 47.13 ± 13.03, 48.01 ± 12.20, 60.79 ± 10.64 and 45.50 ± 12.64 yr, respectively. The average body mass index (BMI) was 22.90 ± 3.40, 23.67 ± 3.66, 28.42 ± 6.93 and 23.33 ± 4.08, respectively.

**Table 1:** Patient demographics.

Group (n)		Group 1 (212)	Group 2 (105)	Group 3 (28)	Group 4 (113)
**Demographics**	Male, n (%)	74 (34.91%)	46 (43.81%)	16 (57.14%)	47 (41.59%)
	Age, yr	47.13±13.03	48.01±12.20	60.79±10.64	45.50±12.64
	BMI, kg/m^2^	22.90±3.40	23.67±3.66	28.42±6.93	23.33±4.08

Group 1: laparoscopic retroperitoneal adrenalectomy; Group 2: robotic retroperitoneal adrenalectomy; Group 3: robotic transperitoneal adrenalectomy; Group 4: laparoscopic retroperitoneal adrenalectomy. BMI, body mass index. Values expressed as mean ± SD.

Preoperatively, all patients underwent routine laboratory test for differential diagnosis of adrenal lesions including but not limited to blood potassium, aldosteronism test, urine and plasma cortisol, plasma catecholamines and urine vanillylmandelic acid. The presence and anatomical characteristics of adrenal masses were confirmed by non-contrast- and contrast-enhanced computed tomography (CT).

### ADRENAL score

**Figure 1:**
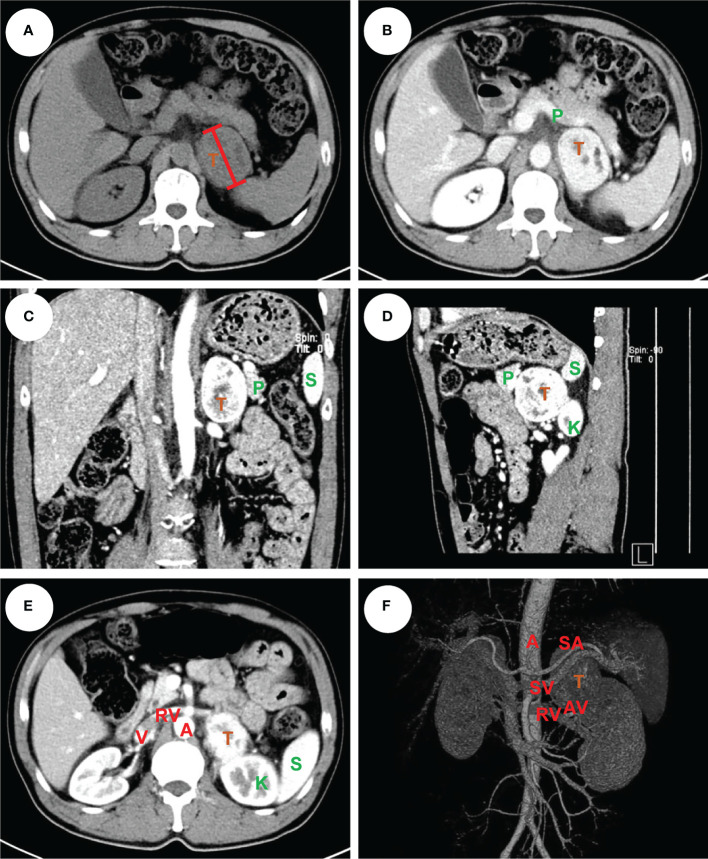
An example of left pheochromocytoma. The male patient had a 6 cm pheochromocytoma arising from the left adrenal gland **(A)** with 110 HU on contrast-enhanced CT **(B)**. A clear tumor boundary was observed on transverse **(B)**, coronal **(C)** and sagittal **(D)** section which revealed a close relationship between the tumor and ipsilateral kidney, spleen and pancreas. A venous phase section **(E)** showed the left renal vein travelled to the vena cava underneath the tumor, which was further confirmed by a 3-D vessel reconstruction based on arterial-venous phase CT scan demonstrating the relative location of the tumor to surrounding major abdominal vessels **(F)**. The tumor was removed robotically *via* retroperitoneal approach. The patient had an ADRENAL score of 2_A_ + 1_D_ + 2_R_ + 2_E_ + 2_N_ + 0_A_ + L_L_ = 9L. T: tumor, P: pancreas, K: kidney, S: spleen, A: aorta, V: (inferior) vena cava, RV: renal vein, SA: splenic artery, SV: splenic vein, AV: adrenal vein.

**Figure 2:**
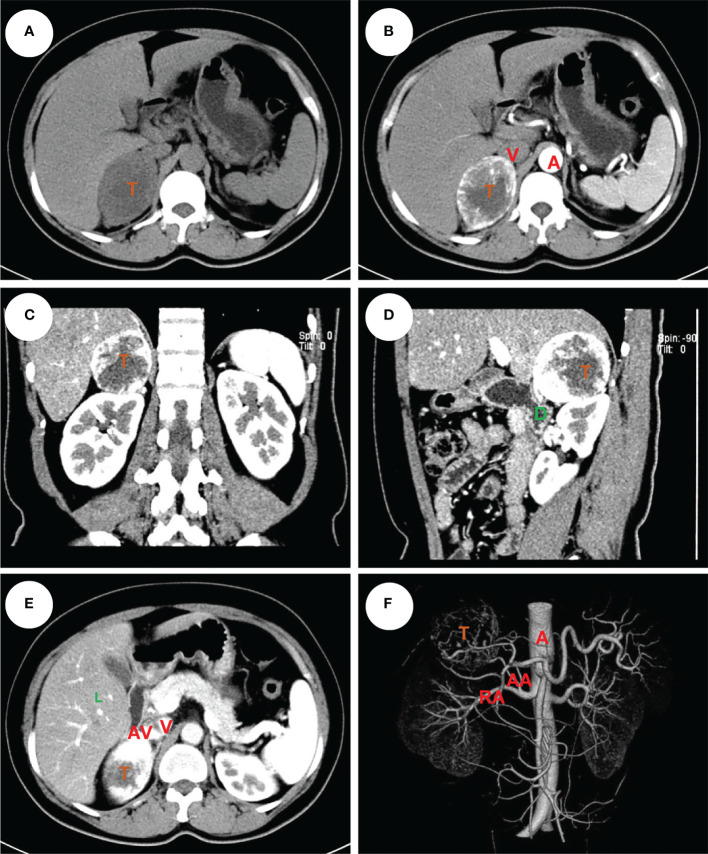
An example of right pheochromocytoma. The female patient had a 6 cm pheochromocytoma arising from the right adrenal gland **(A)** with 120 HU on contrast-enhanced CT **(B)**. A clear tumor boundary was observed on transverse **(B)**, coronal **(C)** and sagittal **(D)** section which revealed a close relationship between the tumor and ipsilateral kidney, right hepatic lobe and duodenum. An arterial-venous phase section **(E)** showed a short adrenal vein draining directly into the vena cava. The 3-D vessel reconstruction based on arterial phase CT scan demonstrated the relative location of the tumor to surrounding major abdominal vessels **(F)**. The tumor was removed robotically *via* retroperitoneal approach. The patients had an ADRENAL score of 2_A_ + 1_D_ + 0_R_ + 2_E_ + 1_N_ + 1_A_ + R_L_ = 7R. T, tumor; L, liver; D, duodenum; A, aorta; V, (inferior) vena cava; RA, renal artery; AA, (middle) adrenal artery; AV, adrenal vein.

The ADRENAL score consists of 7 components that describe critical endocrinological, oncological and anatomical features of an adrenal mass as well as the patient body mass index. Each component in the ADRENAL score is designated by an English letter, forming the acronym ADRENAL: (A)ldosterone/cortisol/catecholamine secretion or suspicion of malignancy based on endocrinological and radiological study, (D)imension (tumor size as the maximal diameter), (R)elationship to adjacent organs, (E)nhancement on computerized tomography, (N)earness of the tumor to major vessels, (A)dipose (patient habitus as body mass index), and a (L)aterality descriptor. Of the 7 components, 6 (A.D.R.E.N.A.) are scored on a 0, 1 or 2-point scale. The 7^th^ descriptor is a suffix which describes the laterality of the mass, either left (l) or right (r). The scoring criteria were described in **Table 2** and illustrated in **Figures 1**, **2** and **Supplemental Figure 1**.

**Table 2:** Components and criteria of the ADRENAL score.

ADRENAL score	0 pt	1 pt	2 pts
**(A)ldosterone/cortisol/** **catecholamine/malignancy**	Non-functional incidentaloma: including myelolipoma, cystic mass, etc	Suspicion of PA, CS	Suspicion of PCC or malignancy
**(D)imension**	≤ 4 cm	> 4 cm but < 7 cm	≥ 7 cm
**(R)elationship to adjacent organs**	A clear tumor boundary without signs of local invasion	< 50% tumor edge with unclear boundary, but no signs of local invasion	≥ 50% tumor edge with unclear boundary, suspicious invasion to adjacent organs
**(E)nhancement on CT scan**	Highest intratumoral CT numbers on contrast-enhanced CT scan < 10 HU	Highest intratumoral CT numbers on contrast-enhanced CT scan ≥ 10 HU but < 85 HU	Highest intratumoral CT numbers on contrast-enhanced CT scan ≥ 85 HU
**(N)earness of the tumor to major vessels**	≥ 7 mm	< 7 mm but not pressing against a major vessel	Pressing against a major vessel
**(A)dipose: BMI (kg/m^2^)**	≤ 25	> 25 but < 30	≥ 30
**(L)aterality**	No points given. Assigned a descriptor of L (left) or R (right).		

PA, primary aldosteronism; CS, Cushing’s syndrome; PCC, pheochromocytoma.

### Data collection and scoring

>In authors’ institute, a team including three urologists, one endocrinologist, one radiologist and one anesthesiologist were assigned to the collection and revision of patient data. Two urologist independently recorded “(D)imension”, “(R)elationship to adjacent organs”, “(E)nhancement on enhanced CT scan”, “(N)earness of the tumor to major vessels”, “(A)dipose: BMI” and “(L)aterality” of the tumor which were verified by the 3^rd^ urologist and an radiologist if any discrepancy noted. The endocrinologist and radiologist were responsible for the evaluation and scoring of “(A)ldosterone/cortisol/catecholamine/malignancy”. Age-adjusted Charlson comorbidity score and ASA (American Society of Anesthesiologists) score of the patients were assessed and reviewed by an urologist with on-line Charlson comorbidity score calculator (http://www.touchcalc.com/calculators/cci_js) and an anesthesiologist, respectively. Tumor characteristics and average ADRENAL score in each group were listed in **Table 3**. In summary, the average tumor size was 3.36 ± 1.95, 4.07 ± 2.72, 5.13 ± 3.94 and 2.97 ± 1.67 cm in Group 1 to 4, respectively. 110 (51.89%), 49 (46.67%), 10 (35.71%) and 58 (51.33%) tumors were located on the left side, respectively. The independently assessed ADRENAL score was 2.52 ± 1.89 (1 to 8), 3.26 ± 2.45 (1 to 9), 4.32 ± 2.50 (2 to 9) and 5.16 ± 1.83 (1 to 9), respectively.

**Table 3:** Tumor characteristics.

		Group 1 (n = 212)	Group 2 (n = 105)	Group 3 (n = 28)	Group 4 (n = 113)
**Tumor** **Characteristics**	Tumor size, cm	3.36±1.95	4.07±2.72	5.13±3.94	2.97±1.67
	Left, n (%)	110 (51.89%)	49 (46.67%)	10 (35.71%)	58 (51.33%)
	Pathology, n (%)				
	Cortical adenoma	146 (68.87%)	63 (60.00%)	11 (39.29%)	81 (71.68%)
	Pheochromocytoma	45 (21.23%)	21 (20.00%)	6 (21.43%)	22 (19.47%)
	Malignancy	6 (2.83%)	2 (1.90%)	9 (32.14%)	4 (3.54%)
	Other	15 (7.08%)	19 (18.10)	2 (7.14%)	6 (5.31%)
**ADRENAL score**		**2.52±1.89**	**3.26±2.45**	**4.32±2.50**	**5.16±1.83**

Group 1: laparoscopic retroperitoneal adrenalectomy; Group 2: robotic retroperitoneal adrenalectomy; Group 3: robotic transperitoneal adrenalectomy; Group 4: laparoscopic retroperitoneal adrenalectomy. ADRENAL score: refer to **Table 2** for details on the scoring system. Values expressed as mean ± SD.

### Statistical analysis

Data are shown as the mean plus or minus standard deviation (SD). Statistical difference in ASA score and age-adjusted Charlson comorbidity score among different ADRENAL score groups were analyzed by one-way ANOVA test. The correlation analysis of the ADRENAL score and each component verse operative time (OT) and estimated blood loss (EBL) was performed by Pearson correlation test. The correlation of the ADRENAL score and OT or EBL was tested in linear regression model, in which an R^2^ of greater than 0.5 was considered a significant correlation. Statistical difference in OT and EBL among Grade I, Grade II and Grade III groups were analyzed by Student t test. Pearson chi-square test was used to calculate the odds ratio. All data were analyzed with the Statistical Package for Social Science Software, v.16.0 (SPSS Inc., Chicago, IL, USA) and a p-value of less than 0.05 was considered statistically significant. Graphs were plotted in GraphPad Prism v.7 (GraphPad Software Inc., La Jolla, CA, USA) or Microsoft Excel 2010 (Microsoft Corp., Redmond, WA, USA).

## Results

### Surgical outcomes

Surgical outcomes of the four groups of patients were listed in **Table 4**. In summary, the average operative time was 130.27 ± 50.30, 149.64 ± 72.64, 208.04 ± 120.65 and 106.96 ± 49.16 min in Group 1 to 4, respectively. To note that the operative time for both laparoscopic and robotic surgery was defined as from the first incision to the closure of all incisions. The average estimated blood loss was 134.96 ± 101.71, 85.14 ± 155.60, 261.60 ± 570.47 and 93.85 ± 153.49 ml, respectively. One case in Group 1, another case in Group 2 and 5 cases in Group 4 were converted to open surgery due to significant adhesion or excessive bleeding. No conversion was observed in Group 3. One case in Group 1, another case in Group 2 and 2 cases in Group 3 were reported to have positive surgical margin. Surgical margin was negative for all cases in Group 4. The average hospital stay was 15.70 ± 6.88, 15.34 ± 4.68, 3.46 ± 3.54 and 15.75 ± 8.18 days, respectively. Overall complication rate was 9.91% (n = 21), 3.81% (n = 4), 7.14% (n = 2) and 25.66% (n = 29), respectively. No patient death was observed during perioperative period in all series. Postoperative pathological study (**Table 3**) revealed cortical adenoma [146 (68.87%), 63 (60.00%), 11 (39.29%) and 81 (71.68%) cases in Group 1 to 4, respectively] as the most common indication, followed by pheochromocytoma [45 (21.23%), 21 (20.00%), 6 (21.43%) and 22 (19.47%), respectively] and other pathologies including cyst, hematoma, angioleiomyolipoma, myelolipoma, and rarely B-cell lymphoma. Malignancy of the adrenal gland was rare [Group 1: 6 (2.83%); Group 2: 2 (1.90%); Group 4: 4 (3.54%)] but accounted for 32.14% (n = 9) in Group 3 due to selection of patients.

**Table 4:** Surgical outcomes.

		Group 1 (n = 212)	Group 2 (n = 105)	Group 3 (n = 28)	Group 4 (n = 113)
**Outcomes**	OT, min	130.27±50.30	149.64±72.64	208.04±120.65	106.96±49.16
	EBL, ml	134.96±101.71	85.14±155.60	261.60±570.47	93.85±153.49
	Conversion, n (%)	1 (0.47%)	1 (0.95%)	0	5 (4.42%)
	PSM, n (%)	1 (0.47%)	1 (0.95%)	2 (7.14%)	0
	LHS, day	15.70±6.88	15.43±4.59	3.46±3.54	15.75±8.18
	Overall Complications, n (%)	21 (9.91%)	4 (3.81%)	2 (7.14%)	29 (25.66%)

Group 1: laparoscopic retroperitoneal adrenalectomy; Group 2: robotic retroperitoneal adrenalectomy; Group 3: robotic transperitoneal adrenalectomy; Group 4: laparoscopic retroperitoneal adrenalectomy. OT, operative time; EBL, estimated blood loss; LHS, length of hospital stays. Values expressed as mean ± SD.

### Validation of the ADRENAL score

The ADRENAL score was applied to all patients according the scoring criteria as demonstrated previously (**Table 2** and **Supplemental Table 1**). Patients were grouped according to ADRENAL score in each series. The variance in age-adjusted Charlson comorbidity score (11) and ASA (American Society of Anesthesiologists) score (16), two reported risk factors of adrenal surgery, was first tested (**Supplemental Figure 2**). Results showed that the difference in comorbidity and ASA score was statistically significant in Group 3 (p = 0.0119), but not in Group 1 (p = 0.4137), Group 2 (p = 0.2251) or Group 4 (p = 0.7208). A significant difference in ASA score was found in Group 4 (p = 0.0019), but not in Group 1 (p = 0.2721), Group 2 (p = 0.7546) or Group 3 (p = 0.2251). Keeping these in mind, we proceeded to validation of correlation between ADRENAL score and OT and EBL, two quantifiable major surgical outcomes.

We first tested the correlation between each of the 7 components in ADRENAL score verse OT and EBL (**Table 5**). Based on the scoring criteria of ADRENAL score, a 0, 1 or 2 points was assigned to tumor function and malignancy (A), tumor size (D), relationship with adjacent organs (R), intratumoral enhancement (E), distance to major vessels (N) and BMI (A). Correlation between the scored value of the above 6 components and the actual value of tumor size (D) and BMI (A) verse OT and EBL was evaluated by Pearson correlation test. Results showed that these 6 components seemed to inconsistently correlate with OT and EBL in all 4 groups. The exact *p* values were listed in **Table 5**. No significant difference was found in either OT or EBL between left and right tumors in either group by Student t test. Due to the selection of patients, we did not include tumors with local invasion in the indication of our laparoscopic adrenalectomy (Group 1). The (R) score was therefore 0 for all cases in Group 1 and cannot be analyzed.

**Table 5:** Correlation analysis of each single components in ADRENAL score, ADRENAL score and a Grade classification verse OT and EBL.

Group (n)	Surgical outcomes	*p* value								
		(A)^c^	(D)^b,c^	(R)^c^	(E)^c^	(N)^c^	(A)^b,c^	(L)^d^	ADRENAL score^c^	Grade classification based on ADRENAL score^e^
Group 1^a^ (212)	OT	NS	0.0014 <0.0001	N/A	0.0012	0.0005	0.0127 NS	NS	<0.0001	0.0006
	EBL	NS	NS NS	N/A	0.0101	NS	NS NS	NS	0.0090	0.0427
Group 2 (105)	OT	NS	0.0069 0.0100	NS	0.0152	NS	NS NS	NS	0.0024	0.0147
	EBL	NS	0.0045 0.0108	<0.0001	0.0018	0.0003	NS NS	NS	0.0004	<0.0001
Group 3 (28)	OT	NS	0.0404 0.0165	0.0026	NS	NS	NS NS	NS	0.0203	0.0404
	EBL	NS	0.0477 0.0120	<0.0001	NS	0.0460	NS NS	NS	0.0072	0.0279
Group 4 (113)	OT	0.0023	0.0160 0.0004	NS	NS	0.0002	NS NS	NS	<0.0001	0.0009
	EBL	0.0121	0.0098 0.0006	NS	0.0126	0.0015	NS NS	NS	<0.0001	0.0080

Group 1: laparoscopic retroperitoneal adrenalectomy; Group 2: robotic retroperitoneal adrenalectomy; Group 3: robotic transperitoneal adrenalectomy; Group 4: laparoscopic retroperitoneal adrenalectomy. OT, operative time; EBL, estimated blood loss; NS, not significant (p > 0.05); N/A, not available. ^a^All tumors in Group 1 had clear boundary without signs of local invasion. Therefore, (R) score is 0 for all cases in Group 1 and cannot be analyzed. ^b^Upper p value: correlation between the scored value of (D) or (A) verse OT or EBL; lower p value: correlation between the actual value of (D) or (A) verse OT or EBL. ^c^Pearson correlation test. ^d^Student t test. ^e^One-way ANOVA.

**Figure 3:**
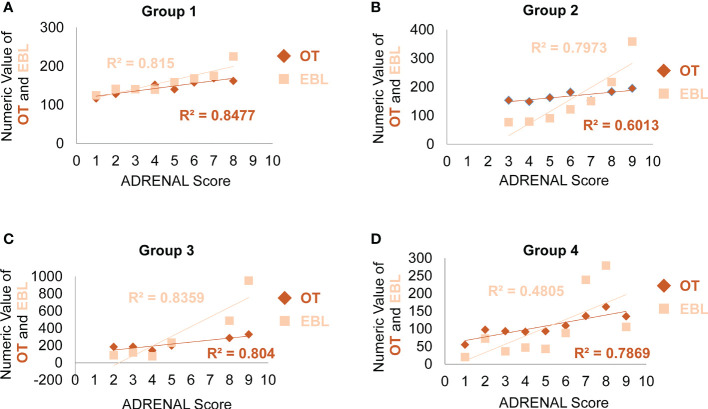
Analysis of the correlation between the ADRENAL score verse OT and EBL. In linear regression model, the correlation between the ADRENAL score and OT or EBL was tested in Group 1 **(A)**, Group 2 **(B)** and Group 3 **(C)** and Group 4 **(D)**. An R^2^ > 0.5 was considered a significant correlation.

Next, we analyzed the correlation between ADRENAL score verse OT and EBL. The correlation between the numeric value of ADRENAL score verse OT and EBL was consistently and statistically significant in all groups by Pearson correlation test (**Table 5**). To further evaluate the value of ADRENAL score in predicting surgical outcomes, we tested ADRENAL score in linear regression model to see whether higher ADRENAL score correlated with longer OT or greater EBL (**Figure 3**). Indeed, the numeric value of ADRENAL score verse OT and EBL fitted well in linear regression model as indicated by an R^2^ of greater than 0.5 (R^2^ was 0.8477 for OT and 0.8150 for EBL in Group 1; 0.6013 for OT and 0.7973 for EBL in Group 2; 0.8040 for OT and 0.8359 for EBL in Group 3; 0.7869 for OT in Group 4). However, the R^2^ for ADRENAL score verse EBL in Group 4 was 0.4805.

### Validation of a grading system based on the ADRENAL score

**Figure 4:**
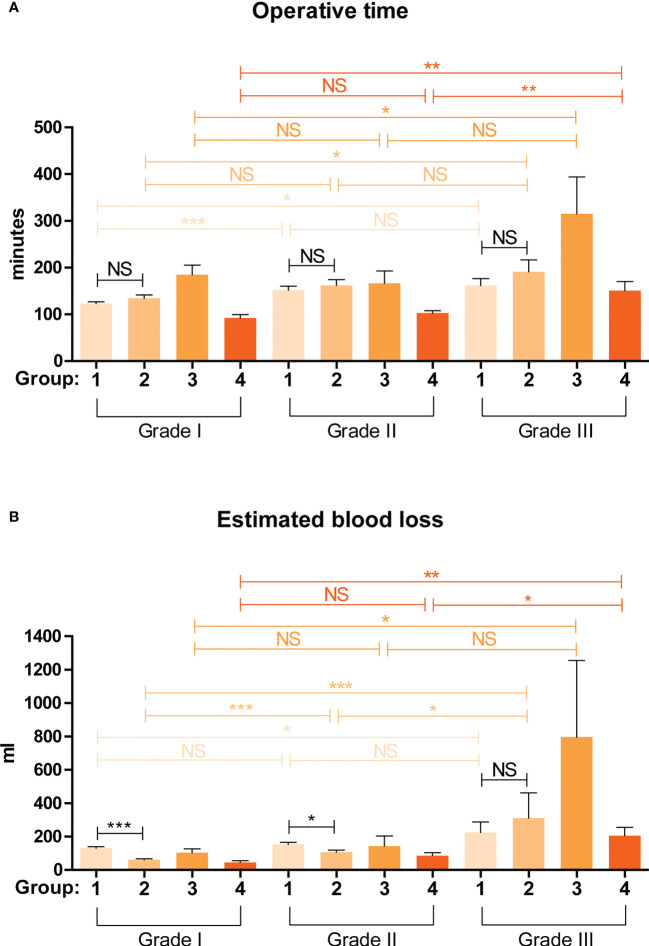
Validation of the predictive value of a grading system based on the ADRENAL score in OT and EBL. A 4-grade classification for adrenal masses was proposed based on the ADRENAL score. Grade 0: ADRENAL score = 0; Grade I: ADRENAL score < 4 (1-3); Grade II: ADRENAL score ≥ 4 but ≤ 7 (4-7); Grade III: ADRENAL score > 7 (8-12). Student t test was performed to analyze the difference in OT and EBL. A p value <0.05 was considered statistically significant. (*p < 0.05; **p < 0.01; ***p < 0.001; NS, not significant).

For more practical use of the ADRENAL score, we proposed a 4-grade classification for adrenal masses based on the ADRENAL score. Grade 0: ADRENAL score = 0; Grade I: ADRENAL score < 4 (1-3); Grade II: ADRENAL score ≥ 4 but ≤ 7 (4-7); Grade III: ADRENAL score > 7 (8-12). Grade 0 adrenal masses or tumors assigned with an ADRENAL score of 0 were not indicated for surgery and managed by watchful waiting in all authors’ institutions, and therefore not included in this study. Percentage of Grade I, Grade II and Grade III tumors in each group were listed in **Supplemental Figure 3**. Student t test was performed to determine any statistical difference in OT or EBL between any two grades in each group (**Figure 4**). Indeed, both OT and EBL in Grade III verse Grade I was consistently statistically significant in all 4 groups (OT: p values for Grade I verse Grade III in Group 1 to Group 4 were 0.0428, 0.0116, 0.037 and 0.0033, respectively; EBL: 0.0322, < 0.0001, 0.0236 and 0.0013, respectively). However, OT or EBL of Grade I verse Grade II or Grade II verse Grade III in the 4 groups was not consistently statistically significant (OT: p values for Grade I verse Grade II in Group 1 to Group 4 were 0.0004, NS, NS and NS, respectively; Grade II verse Grade III: NS, NS, NS and 0.0011, respectively; EBL: p values for Grade I verse Grade II in Group 1 to Group 4 were NS, 0.0003, NS and NS, respectively; Grade II verse Grade III: NS, 0.024, NS and 0.013, respectively; NS: not significant), despite the fact that higher grade correlated with longer OT and larger EBL in all 4 groups (**Supplemental Tables 2**, **3**).

Surgeries of Group 1 and Group 2 patients were performed by the same surgical team using conventional laparoscopy and da Vinci robotic system, respectively, both *via* retroperitoneal approach. By comparing the surgical outcomes of these two groups stratified by the 4-grade classification based on the ADRENAL score, which we believe was more reasonable than an overall comparison, we could determine whether robotic surgery was indeed superior to laparoscopic surgery eliminating any bias from different surgical techniques and approaches. We first did a non-stratified analysis of the difference in overall OT and EBL between Group 1 (laparoscopic) and Group 2 (robotic). Robotic surgery seemed to take a slightly longer time than laparoscopic surgery (146.21 ± 64.75 vs 130.89 ± 50.46 min) but with a significant *p* value of 0.0215, while the blood loss was significantly lower in robotic group than laparoscopic group (101.06 ± 138.88 vs 151.69 ± 94.14 ml, *p* = 0.0020). Stratified by the 4-grade classification based on the ADRENAL score (**Figure 4**), no significant difference was found in OT between robotic and laparoscopic group in Grade I, Grade II, nor Grade III tumors. However, robotic surgery seemed to correlate with significantly lower EBL than laparoscopic group in both Grade I and Grade II tumors (p < 0.0001 and p = 0.0129, respectively), but not in Grade III tumors.

### Complication, conversion, transfusion and length of hospital stay

In terms of perioperative complications (Group 1: number of complications/number of cases = 21/212, overall complication rate: 9.91%; Group 2: 4/105, 3.81%; Group 3: 2/28, 7.14%; Group 4: 28/113, 24.78%), most were Clavien-Dindo Grade I including fever and vomiting managed with antipyretics and antiemetics, respectively. 9 patients in Group 1, 1 patient in Group 2 and 2 patients in Group 3 had postoperative infection and managed with antibiotics. 1 patient in Group 1, 2 patients in Group 2 and 1 patient in Group 2 had abnormal blood pressure and managed with vasoactive drugs accordingly. 1 patient in Group 2 and 1 patient in Group 3 converted to open surgery due to excessive adhesion and received blood transfusion. No Grade III to Grade V complication was encountered in patients included in this study. Overall, patients with Grade II and Grade III tumor seemed to have a higher, but not statistical significant, risk of perioperative complication rate than those bearing Grade I tumor (**Supplemental Table 4**).

Conversion to open surgery was only observed in 1 Grade II case in Group 1, 1 Grade III case in Group 2, 1 Grade III case in Group 3, 2 Grade II cases and 3 Grade III cases in Group 4, due to significant adhesion (**Supplemental Table 5**). All conversion was observed in Grade II or Grade III tumors, while none in Grade I tumors in all 4 groups. 2 Grade II cases in Group 1, 1 Grade III case in Group 2, 1 Grade III case in Group 3, 1 Grade I case, 3 Grade II cases and 3 Grade III cases in Group 4 received intraoperative transfusion due to excessive bleeding (**Supplemental Table 6**).

Length of hospital stay was listed in **Supplemental Table 7**. To note that the length of hospital stay in Group 1, 2 and 4 (Chinese hospitals), which was defined as from the first day of administration to the day of discharge, appeared to be significantly longer than that in Group 3 (US hospital) due to different policies for inpatient management. Results showed that patients bearing Grade III tumor had significantly prolonged hospital stay in Group 2 and Group 4, compared with patients with Grade I tumor in each respective group.

## Discussion

Adrenal tumor is a common indication of surgery in urology. The functional status and suspicion of malignancy (fast growing, large size, signs of local invasion or metastatic lesion) are considered to be the two most important factors when deciding if an adrenal tumor should be removed. As far as surgery is concerned, the functional status (11), pheochromocytoma (12) and malignancy (13), tumor size (14) and BMI (15) are all documented as risk factors. In particular, while most adrenal tumors are small, large tumor still spans a significant portion (136 out of 458 adrenal tumors included in this study were greater than 4 cm, among which 37 tumors were greater than 7 cm). As such, the relationship between the tumor and surrounding structures (large vessels and organs) may pose a unique challenge and needs to be reviewed before surgery.

It is the goal of both the patient and the surgeon to ensure that the adrenal tumor can be safely and completely removed. Achieving this goal relies on an accurate assessment of the patient’s risk before surgery. After all, high-risk patients are more likely to be in danger during surgery. However, due to the complex and diverse risk factors affecting surgery, how to establish an evaluation system that incorporates various factors is a problem for urologists to think about. Some relevant evaluation systems, such as the weiss scoring system (17), the pheochromocytoma of the adrenal gland scaled score (PASS), the grading system for adrenal pheochromocytoma and paraganglioma (GAPP) (18, 19), and the utrecht score (20), have some reference significance, but they are mostly based on postoperative pathology to predict expected survival rather than surgical risk assessment. Only Caiazzo (21) provided an adrenalectomy risk score. There has been little research on surgical risk assessment systems for patients with adrenal tumors, which is why we conducted this study.

In this study, we proposed an original and standardized preoperative scoring system, designated as the ADRENAL score, which integrates these established risk factors and tumor anatomy for adrenal masses. This scoring system quantitatively describes the nature and anatomical characteristics of adrenal masses based on multidisciplinary assessment that are routinely performed in the clinical practice, looking to provide an overall estimation of the clinical significance of the tumor and the risk of surgery. In the clinical practice, endocrinologists and urologists may be assisted by this 0 to 12 scoring system coupled with a laterality indicator when making any clinical decision on the indication of surgery, preoperative preparation and assessment, intraoperative precautions, postoperative management and follow-up. As far as surgery is concerned, the ADRENAL score has been shown in this study to predict the operative time and estimated blood loss in minimally invasive adrenal surgeries. By comparative analysis, our data suggested that the integrated scoring system may be more accurate and comprehensive in predicting operative time (OT) and estimated blood loss (EBL) than a single risk factor. ADRENAL score seemed to be positively, but not statistical significantly related with complication, transfusion and conversion. Larger sample size is required to establish the possible correlation. Further, patients with higher ADRENAL score were subjected to significantly longer hospital stay, which may be explained by higher (but not statistically significant) complication, transfusion and conversion rate, and a longer time required for a definitive diagnosis before surgery (for Group 1, 2 and 4).

Our current data suggested a possible predictive role of ADRENAL score in various surgical outcomes. As such, besides preoperative risk assessment, the ADRENAL score may serve as a valid grading system in risk-stratified designing of adrenalectomy training programs for the training of young doctors and safety of patients. For conducting clinical research, the ADRENAL score could be a valuable tool for more scientific evaluation of a surgeon’s learning curve, and comparative studies on adrenal surgeries *via* different techniques and approaches.

### Design of study

In this study, we included 4 groups of patients treated with laparoscopic or robotic adrenalectomy *via* retroperitoneal or transperitoneal approach, aiming to achieve an unbiased validation of the ADRENAL score. To improve the quality of the analysis, doctors involved in the data collection and scoring process were all blinded to the final outcome of statistical analysis; and a single or the same group of doctors was assigned to the scoring of a specific component in the ADRENAL score for each group. The rational of including the 7 components in the ADRENAL score was given in **Supplemental Materials**.

### Limitations of study

One potential limitation of this study is the lack of stratified evaluation of complications based on Clavien-Dindo classification (22). Further studies are required to establish a possible correlation between the ADRENAL score and the grade of complications. We also noted a significant variance (p = 0.0119) in age-adjusted Charlson comorbidity score among patients assigned with different ADRENAL score from Group 3. As such, the possible interference of patients comorbidity cannot be ruled out in all statistical analysis in Group 3. Another limitation is that we did not include open surgery in our study, which is still the standard of care for adrenal malignancy. Further external validation of the ADRENAL score is required.

## Conclusions

The ADRENAL score of adrenal mass is a comprehensive scoring system which integrates established risk factors and the most important anatomical features of the tumor. It can be assessed retrospectively and prospectively by routinely performed endocrinological, oncological and radiological studies of adrenal mass. Our study demonstrated that the ADRENAL score seems to correlate with surgical outcomes. Therefore, it may provide a common reference for adrenal surgery training program, preoperative risk assessment and stratified comparative analysis of adrenal surgeries *via* different techniques and approaches. Validation of this system in other institutions is currently on-going.

## Data availability statement

The original contributions presented in the study are included in the article/**Supplementary Material**. Further inquiries can be directed to the corresponding authors.

## Ethics statement

The studies involving human participants were reviewed and approved by The Ethical Committee of The First Affiliated Hospital of Nanchang University. The patients/participants provided their written informed consent to participate in this study.

## Author contributions

Conceived and designed the experiments: XZ, BF, DE, and GW. Console surgeon: DE, GW and YD. Collected and analyzed the data: XL, WL, CZ, YX, HG, LZ, EL, JK. Wrote the paper: XZ and XL. All authors contributed to the article and approved the submitted version.

## Funding

Fund programs: Key Research and Development Program of Jiangxi Province (20171ACB20029 to XZ).

## Conflict of interest

The authors declare that the research was conducted in the absence of any commercial or financial relationships that could be construed as a potential conflict of interest.

## Publisher’s note

All claims expressed in this article are solely those of the authors and do not necessarily represent those of their affiliated organizations, or those of the publisher, the editors and the reviewers. Any product that may be evaluated in this article, or claim that may be made by its manufacturer, is not guaranteed or endorsed by the publisher.
