# Supplementary figures and images for: The ADRENAL score: A comprehensive scoring system for standardized evaluation of adrenal tumor
Source: Front Endocrinol (Lausanne). 2022 Nov 24;13:1073082. doi: 10.3389/fendo.2022.1073082 (PMC9730271; doi:10.3389/fendo.2022.1073082)

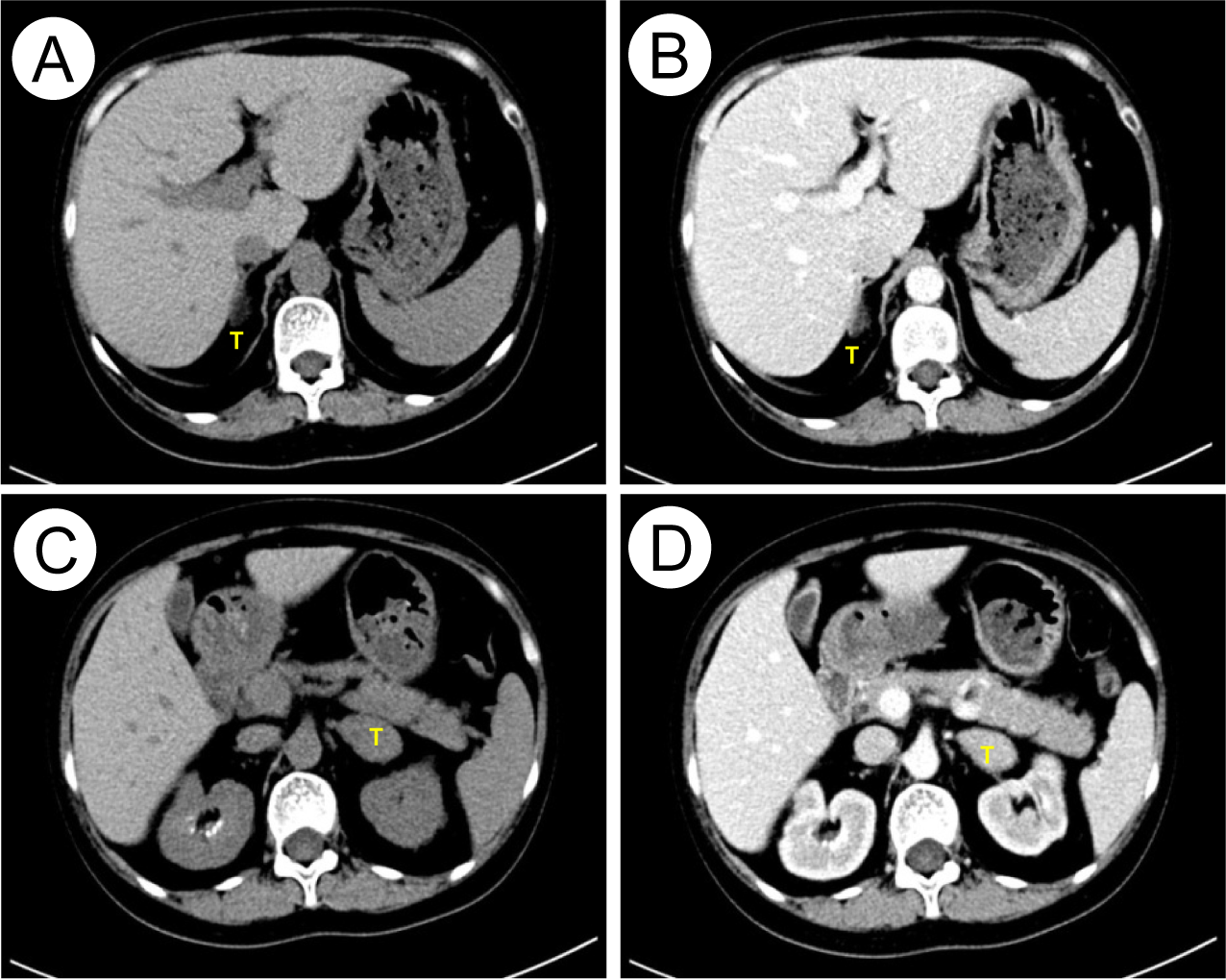

Supplement: Supplementary file 1 [file Image_1.tif]

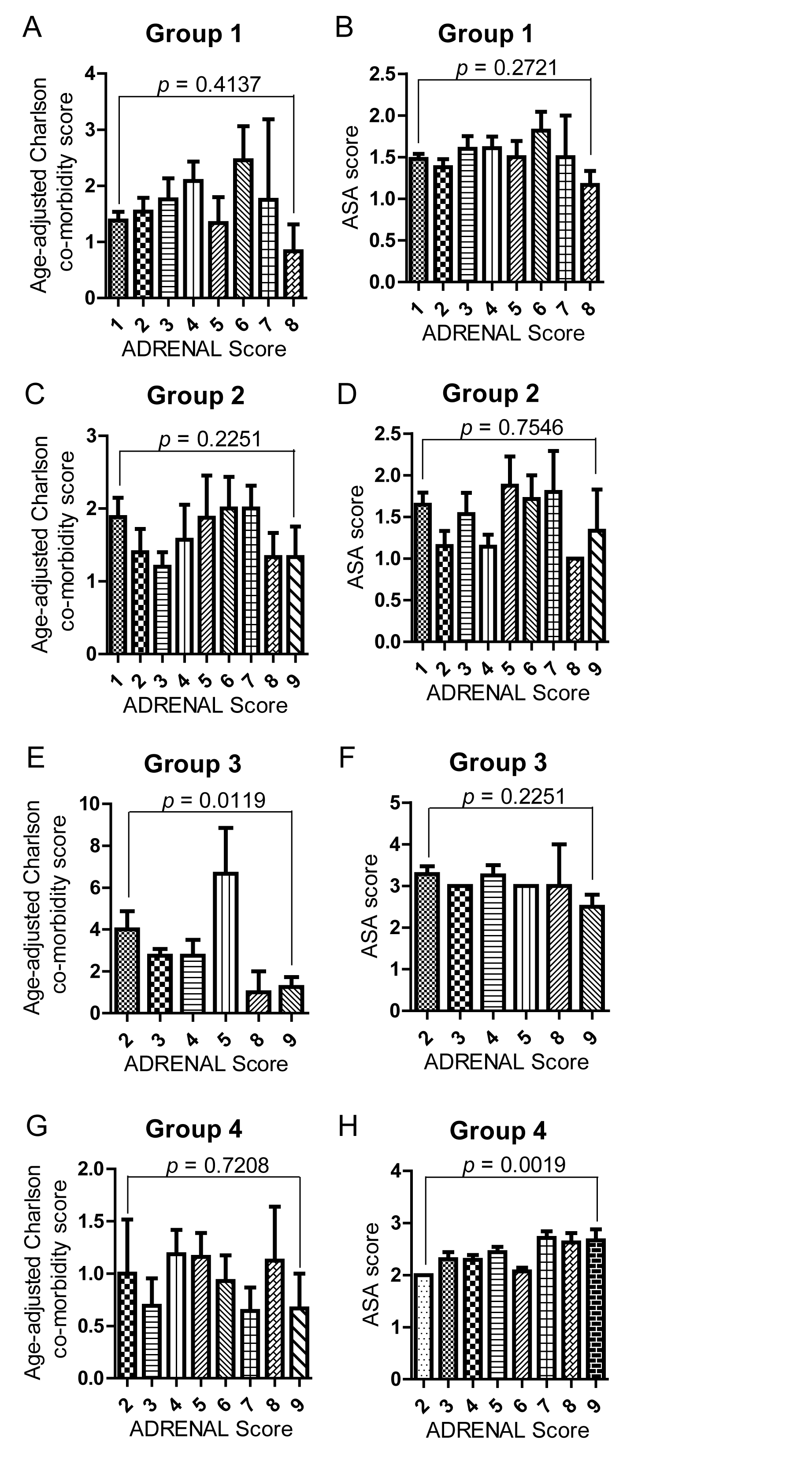

Supplement: Supplementary file 2 [file Image_2.tif]

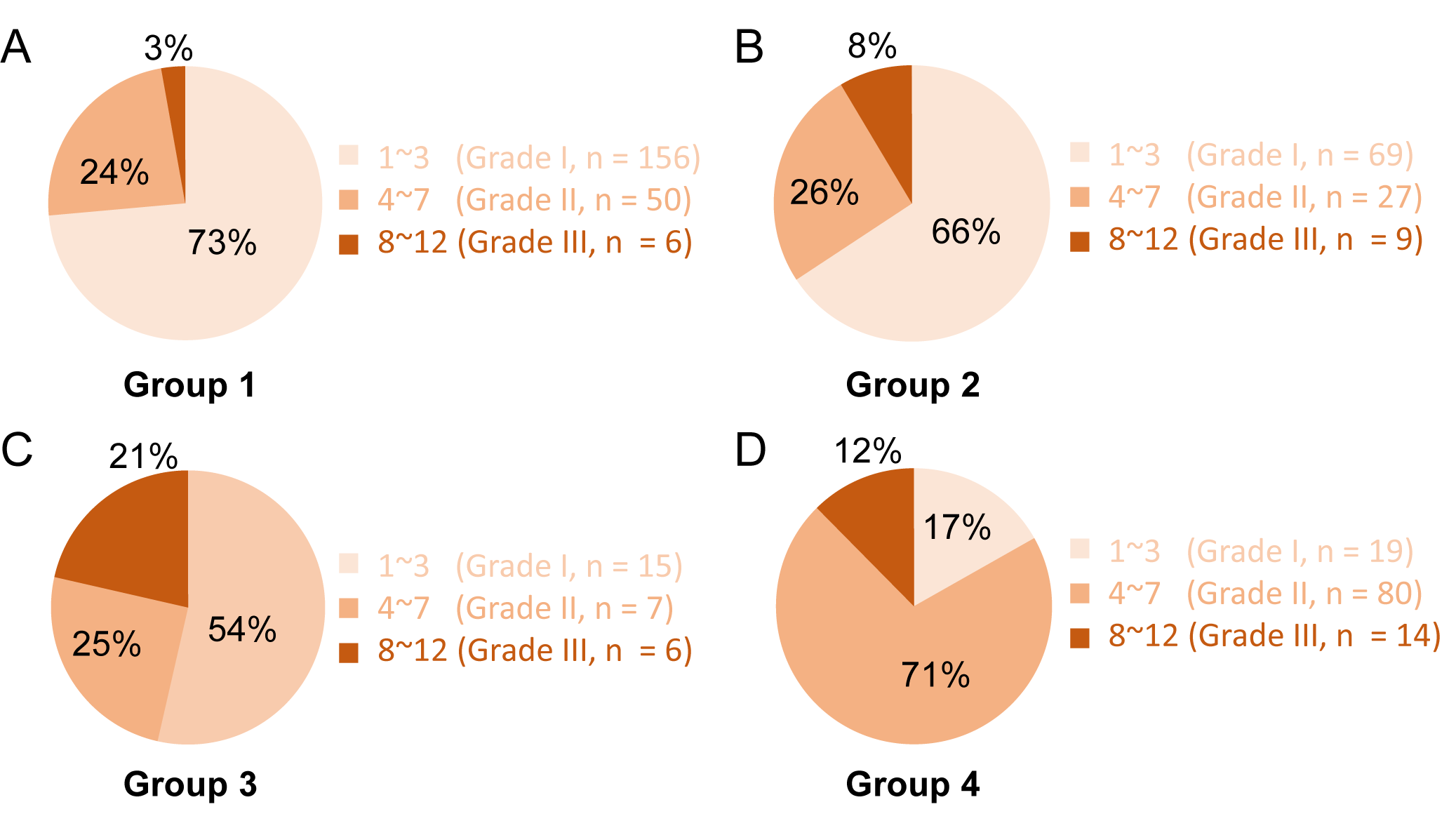

Supplement: Supplementary file 3 [file Image_3.tif]
